# Comparison of Cardiac Autonomic Function in Type 2 Spinocerebellar Ataxia With Normal Control Using Heart Rate Variability as a Tool: A Cross-Sectional Study in Eastern India

**DOI:** 10.7759/cureus.20058

**Published:** 2021-11-30

**Authors:** Laxman K Senapati, Sudipta Patnaik, Priyadarsini Samanta, Sambit P Kar, Santosh Dash, Jayanti Mishra

**Affiliations:** 1 Department of Anesthesia, Kalinga Institute of Medical Sciences, Kalinga Institute of Industrial Technology (KIIT, deemed to be University), Bhubaneswar, IND; 2 Department of Physiology, Sriram Chandra Bhanja Medical College, Utkal University, Bhubaneswar, IND; 3 Department of Physiology, Kalinga Institute of Medical Sciences, Kalinga Institute of Industrial Technology (KIIT, deemed to be University), Bhubaneswar, IND; 4 Research, School of Electronics Engineering, Kalinga Institute of Industrial Technology (KIIT, deemed to be University), Bhubaneswar, IND; 5 Department of Neurology, Kalinga Institute of Medical Sciences, Kalinga Institute of Industrial Technology (KIIT, deemed to be University), Bhubaneswar, IND

**Keywords:** sympathetic tone, parasympathetic tone, cardiac autonomic function, heart rate variability, spinocerebellar ataxia

## Abstract

Background: Spinocerebellar ataxia (SCA) is a disease that refers to a category of inherited ataxias that are characterized by degenerative alterations in the cerebellum, pons, and spinocerebellar tracts. There are several different varieties of SCA and they are classified based on the mutant (altered) gene that causes the disease.

Objective: To analyze the cardiovascular autonomic regulation in patients with type-2 spinocerebellar ataxia (SCA-2) from the heart rate variability (HRV) of 20 minutes resting electrocardiogram (ECG) and compare with the age and gender-matched controls.

Materials and methods: HRV of 27 type-2 spinocerebellar ataxia patients was calculated offline from the resting ECG recording and compared with 23 age and gender-matched controls. The HRV was analyzed by HRV software module MLS 310. The frequency and time domain parameters were computed and compared.

Result: Type-2 spinocerebellar ataxia patients have significantly low HRV and parasympathetic activity at rest compared to normal control. The total power in SCA-2 is 13491.63 ± 7660.77 ms^2^ and the normal control is 21784.76 ± 11008.67 ms^2^. High-frequency power (HF) which is a marker of parasympathetic activity in SCA-2 is 3823.1 ± 364 ms^2^ and in normal control is 9006.1 ± 920.64 ms^2^. The standard deviation of all NN intervals (SDNN), the square root of the mean-squared differences of successive intervals (RMSSD), spectral interval, and delta NN is significantly low in SCA-2.

Conclusion: There is decreased parasympathetic tone and low HRV in SCA-2 as compared to normal controls.

## Introduction

Spinocerebellar ataxias (SCAs) are a heterogeneous set of progressive neurological illnesses marked by dysequilibrium, increasing loss of gait and limb coordination, and speech and eye movement problems. Spinocerebellar ataxias can occur as a familial form or as a spontaneous, nongenetic disorder. Most forms of cerebellar ataxias are genetic in origin that is autosomal dominant, recessive, or X-linked (SCA-1, SCA-2, or SCA-3). The dominant versions are less severe than the recessive ones. The most basic neurological symptom is ataxia. Ataxia is caused by a lesion of the cerebellum and its immediate connected circuits, proprioceptive sensory pathways, or the vestibular system [1]. The brainstem, spinal cord, and thalamus all have substantial connections with the cerebellum. Of all the neurological signs that develop after cerebellar loss, ataxia or a lack of coordination of gait or limb movements is the most distinctive to this portion of the brain. Spinocerebellar ataxia type-2 (SCA-2) is the commonest worldwide and mostly in India and has an autosomal dominant inheritance where translated protein, Ataxin-2 causes neuronal apoptotic cell death of cerebellum, pons, hypothalamus, and spinal cord related to autonomic control [2-4]. The genetic etiology of SCA-2 is due to a CAG trinucleotide repeat expansion in the gene on chromosome 12q23-24.1 [5,6]. Cerebellar ataxia is a degenerative condition that, while not lethal, can put an undue strain on the heart. Autonomic failure and moderate Parkinsonism occur in some of the patients. Autonomic nervous system (ANS, vagal) dysfunction is emerging as the second commonest type of spinocerebellar ataxia [7].

The cerebellum governs baroreceptor sensitivity and other autonomic processes by modulation of both the sympathetic and parasympathetic systems, and the fastigial nucleus generally exerts an atonic inhibitory influence on cardiac parasympathetic tone [8]. Cerebellum has a role in cerebral vasomotor control. As a result, clinically noticeable disruption of autonomic function may be linked to cerebellar diseases and, in certain cases, to cerebellar circuits [9,10]. The kind and level of autonomic involvement in cerebellar ataxia have been determined by several researchers, resulting in inconsistent reports.

Heart rate variability (HRV) has evolved into an effective tool for assessing the balance between the sympathetic and parasympathetic nervous systems of the ANS. HRV is a basic, non-invasive, consistent, and inexpensive test for assessing cardiovascular autonomic status in ANS dysfunction patients [11,12]. SCA patients have an increased number of clinical autonomic symptoms and lower HRV indices which have previously been reported in different types of SCA [12,13]. HRV components measure the severity of autonomic impairment rather than the level of autonomic tone. One of the most promising measures of autonomic function is HRV. Beat-to-beat cardiovascular control is quantified using power spectral analysis of heart rate fluctuations. Although cardiac automaticity is built into many pacemaker tissues, the autonomic system is in charge of heart rate and rhythm. The beat-to-beat regulatory mechanisms are fine-tuned during resting conditions, as seen by the RR interval fluctuations. Computer-assisted blood pressure and HRV measurement has been utilized to analyze the dynamic aspects of autonomic regulation of CVS in the last two to three decades [14-21].

The aim of the present study is to evaluate the impact of ataxia on the autonomic regulation of CVS by analyzing HRV by utilizing a resting electrocardiogram (ECG). Autonomic nervous system involvement in patients with SCA has rarely been studied and has shown conflicting results.

## Materials and methods

Subjects

All the consecutive cases of genetically confirmed spinocerebellar ataxia type-2, who came to neurology OPD from January to June 2016 were included in the study. We got 27 cases (10 females and 17 males) of spinocerebellar ataxia type-2, with a mean age of 32.59±13.93 years in the study group. A detailed neurological examination was done and the severity of ataxia was graded by a neurologist using International Cooperative Ataxia Rating Scale (ICARS) [22]. There were 23 age-matched normal subjects (11 females and 12 males) of mean age 33.60±11.60 years, who were accompanying the SCA-2 patients. There was no evidence of hypertension, ischemic heart disease, diabetes, chronic obstructive pulmonary disease, or central nervous system disease in the study as well as in the control group. None of the participants were taking any medications that could affect their cardiovascular or nervous systems. After a thorough explanation of the procedure to patients or relatives, as well as control subjects, they gave their written informed consent for the test.

The patients were recruited from S.C.B (Sriram Chandra Bhanja) Medical College, Cuttack, and KIMS (Kalinga Institute of Medical Sciences), Bhubaneswar. The study was conducted in the Department of Physiology, KIMS, Bhubaneswar, Odisha. The study was approved by the Institute Ethics Committee of KIMS, KIIT University, (Ref. No. KIMS/KIIT/IEC/011/2014). The study procedure follows the declaration of Helsinki.

Process of measuring HRV

The patient was given a thorough explanation of the procedure and told to relax. An attendant of the same gender was present in the test room. All of the metallic ornaments had been removed. The patients were asked to refrain from smoking and drinking coffee for at least two hours before the study, which began at 10 a.m. They were also instructed to consume a light breakfast. During the test, no physical handling of the patient was allowed. The lead-II ECG was recorded for 20 min. The 16-channel data acquisition system; Power Lab, Bella Vista, NSW, Australia with chart software was used to study all of the ataxia patients and normal controls. An analog-to-digital converter was used to transport the ECG signals to a computer, where they were processed offline using an HRV analysis program (MLS 310 HRV module). To evaluate and display RR intervals classed as normal, ectopic, or artifact, a threshold detector was employed to detect the R wave in raw ECG data. The parameters in the time domain and frequency domain were computed automatically. The ML 132 Bio amplifier and MLA 1340 subject cable were used to record the ECG. In the right and left midclavicular lines, MLA 1010 disposable ECG electrodes were placed infraclavicularly. The sampling rate was 1024 per second.

Measured HRV parameters

The time-domain parameters which were measured to perform analysis are detailed below in Table 1.

**Table 1:** The time-domain measures of heart rate variability NN: normal-to-normal, SDNN: standard deviation of all NN intervals, RMSSD: square root of the mean-squared differences of successive intervals, NN50: number of pairs of adjacent NN intervals differing by more than 50 ms, SD of Delta NN: SD of the difference between adjacent NN

Variable (ms)	Statistical measures
Mean NN	The mean of the normalized RR intervals
SDNN	The standard deviation of all NN intervals
RMSSD	The square root of the mean of the sum of the squares of differences between adjacent NN intervals
NN50	Number of pairs of adjacent NN intervals differing by more than 50 ms in the entire recording
SD of delta NN	The SD of the difference between adjacent NN intervals
Ratio	The ratio SDNN/RMSSD

The frequency-domain parameters were noted using fast Fourier transformation algorithms (FFT). The parameters are detailed in Table 2 [12].

**Table 2:** Frequency domain measures of heart rate variability using FFT VLF: very low frequency, LF: low-frequency, HF: high-frequency, FFT: fast Fourier transformation

Variable (ms^2^)	Description	Frequency range
Total power	The total power in the spectrum for the current analysis region	
VLF	Power in the VLF range	≤0.04 Hz
LF	Power in the low-frequency range	0.04 –0.15 Hz
HF	Power in the high-frequency range	0.15–0.4 Hz
LF/HF	The ratio of LF/HF	

Statistical analysis

The continuous data were presented as mean±SD. All the abbreviations followed the recommendation of the European Society of Cardiology and the North American Society of Pacing and Electrophysiology [14]. The mean and standard deviation of each parameter was calculated and compared with that obtained from normal subjects. All the data were normally distributed as tested by the Shapiro-Wilk test. Statistical method, the unpaired t-test was used to assess the difference between patients and controls for different parameters of HRV at rest. Statistical significance for all analyses was defined as P < 0.05. The analysis of the dataset was carried out in the IBM SPSS software version.20 (IBM Corp., Armonk, NY) using a system running on Windows 7 operating system.

## Results

The demographic parameters are presented in Table 3. The mean age of SCA-2 patients was 32.59 ± 13.93 years and the mean age of controls was 33.60 ± 11.60 years. There were 17 males and 10 females in the SCA-2 group whereas 12 males and 11 females were in the control group. The mean height of SCA-2 patients was 158.62 ± 16.03 cm and that of controls was 164.47 ± 9.39 cm. The mean weight of SCA-2 and controls were 58.63 ± 13.56 kg and 62.26 ± 10.85 kg, respectively. The age, gender, height, and weight were comparable in both groups. The mean age of onset of ataxia was 25.96 ± 15.40 years and the mean duration of disease was 6.14 ± 5.65 years in the SCA-2 patients. The ICARS score was 29.4 ± 9.98 in SCA-2 patients.

**Table 3:** Demographic Parameters ICARS: International Cooperative Ataxia Rating Scale

	SCA-2 (n=27)	Control (n=23)	p-value
Age (years)	32.59 ± 13.93	33.60 ± 11.60	0.78
Mean height (cm)	158.62 ± 16.03	164.47 ± 9.39	0.13
Mean weight (kg)	58.63 ± 13.56	62.26 ± 10.85	0.305
Male/female	17:10	12:11	
Age of onset of ataxia (years)	25.96 ± 15.40	---	
Duration of disease (years)	6.14 ± 5.65	---	
ICARS score	29.4 ± 9.98	---	

The time-domain parameters are presented in Table 4. The mean NN, SDNN, SD of delta NN, RMSSD, and NN 50 were significantly less in the SCA-2 group as compared to controls. The ratio of SDNN and RMSSD was increased in the SCA-2 group, which was statistically insignificant.

**Table 4:** Time domain analysis (p<0.05, statistically significant) Mean NN: mean of normalized RR intervals; SDNN: standard deviation of all NN intervals; SD of delta NN: SD of the difference between adjacent NN; Ratio: ratio SDNN/RMSSD; RMSSD: square root of the mean-squared differences of successive intervals; NN50: number of pairs of adjacent NN intervals differing by more than 50 ms

Parameters	Control (n=23)	SCA-2 (n=27)	P-value
Mean NN (ms)	821.84 ± 172.01	657.40 ± 146.25	0.0007
SD NN (ms)	195.2 ± 12	112.11 ± 62.73	<0.0001
SD of delta NN	235.44 ± 160.42	120.22 ± 70.64	0.0015
Ratio	0.93 ± 0.32	1.10 ± 1.07	0.4
RMSSD (ms)	227.95 ± 114.14	120.06 ± 70.57	0.0002
NN50	263.37 ± 141.80	111.7 ± 91.7	<0.0001
Mean spectral RR	821.71 ± 171	557.69 ± 142.76	<0.0001

The frequency-domain parameters are presented in Table 5. The total power, low-frequency power (LF), and high-frequency power (HF) were significantly reduced in the ataxic group as compared to controls. But there were no significant reductions in the very low-frequency power (VLF) in SCA-2. The ratio of LF and HF was found to be increased significantly in the SCA-2 group.

**Table 5:** Frequency domain analysis (p<0.05, statistically significant) VLF: very low frequency; LF: low frequency; HF: high frequency

Parameters	Control (n=23)	SCA-2 (n=27)	P-value
Total power	21784.76 ± 11008.67	13491.63 ± 7660.77	0.003
VLF	6250.8 ± 1143.62	5758.6 ± 1071.04	0.123
LF	6503.6 ± 630.21	3910.9 ± 485.4	<0.0001
HF	9006.1 ± 920.64	3823.1 ± 364	<0.0001
LF/HF	0.90 ± 0.51	1.66 ± 1.5	0.02

Hence, the significantly low SDNN, SD of delta NN, RMSSD, total power, LF, and HF power in the SCA-2 group in comparison to normal control reflects a lower HRV. Low RMSSD, NN50, and HF indicate impaired parasympathetic modulation of heart rate.

## Discussion

There is a paucity of studies showing HRV in SCA. In all varieties of SCA, the cause of autonomic dysfunction is most likely due to peripheral polyneuropathy besides the action of central autonomic centers [4,23-26]. The time-domain parameters in our study, such as SDNN, NN50, RMSSD are significantly low in SCA-2. The SDNN is a measure of overall activity whereas NN50 and RMSSD are measures of parasympathetic activity. The frequency-domain parameters such as total power, HF power, LF power are measured in short-term HRV recording. The TP, LF, and HF in our study are also significantly reduced. VLF is decreased but not significantly. HF denotes parasympathetic activity but LF denotes both sympathetic and parasympathetic activity. VLF signifies sympathovagal balance.

The HRV analysis had demonstrated a lower HRV in ataxia patients as evident from the significant decrease in RMSSD, NN50, total power, LF, and HF power. This finding correlates with the findings of Pradhan et al. [12] and Montes-Brown et al. [20]. Decreased HRV reflects impaired parasympathetic autonomic modulation of the heart and the converse is true [16]. Similarly, a decrease in high frequency is due to decreased parasympathetic activity or vagal tone in spinocerebellar ataxia, which concurs with the findings of Ernst [27]. Choudhury identified autonomic dysfunction in movement disorders [28]. According to Sanchez et al., SCA regulates autonomic function through the involvement of the peripheral nervous system and the central nervous system [29]. Spinocerebellar degeneration is frequently accompanied by autonomic failure, according to Azuma et al. [30]. Yazawa et al. found severe reductions in HRV parameters in olivopontocerebellar ataxia and were caused by considerable reductions in sympathetic and parasympathetic nerve activity [31]. According to Yeh et al., autonomic dysfunction is prevalent in spinocerebellar ataxia [11]. The findings of all the above studies concur with our study.

In autosomal dominant cerebellar ataxia, there is no significant reduction in parasympathetic response and a substantial drop in sympathetic pressor response according to Singh et al. [32]. Ingall and McLeod discovered that an increase in resting supine heart rate was caused by cardiac anomalies rather than autonomic dysfunction [33], which does not correlate with our study.

There is a shift of neurocardiac balance toward sympathetic predominance in spinocerebellar ataxia compared with controls at rest. Enhanced vagal influence on the heart, leads to an enhanced barosensitivity, which is a powerful source of vagal afferent input to the CNS and one of the most important physiological mechanisms affecting efferent cardiac vagal activity [18].

SCA is differentiated by advanced degeneration and later loss of neurons along with reactive gliosis, and the clinical symptoms are specific to the loci of the neurons which are degenerated and lost. The spinal neurons are mostly affected by degeneration while other neurons are less affected. The cerebellar function is deranged progressively in most of the SCA, characterized by ascending instability of gait, limb movement in coordination with impaired skilled movements. Other neuronal systems affected in SCA are the corticospinal tract, basal ganglia, and autonomic nuclei of the brain and spinal cord [18,19]. The unusually low parasympathetic activity may be explained by the peripheral neuronopathy, axonopathy, and involvement of central autonomic centers.

## Conclusions

Our study found low HRV parameters including the time domain and frequency domain in type-2 spinocerebellar ataxia patients, which shows that there is the involvement of parasympathetic as well as sympathetic autonomic nerves in these patients. But the parasympathetic involvement is more severe than sympathetic involvement. The observed HRV changes are due to severe vagal nerve degeneration resulting in cardiovascular autonomic neuropathy in patients with SCA-2. However, we should not reject the neuronal denervation of sympathetic autonomic nerve fibers, which was quantitatively assessed in the present study. The present study shows that the investigation of the time domain and frequency domain of HRV is a powerful tool in the management of patients with limited and impaired autonomic control of the cardiovascular system. The low HRV is a diagnostic marker for the autonomic involvement in spinocerebellar ataxia. So, we recommend that the neurologists should advise the SCA patients to undergo a complete cardiovascular autonomic assessment including HRV, thereby preventing the severity of cardiovascular morbidity and mortality in SCA patients. Further longitudinal studies are required taking more sample size. The percentage of autonomic involvement using HRV in various types of SCA can also be ascertained in future studies.
